# Stress and Occupational Burnout of Nurses Working with COVID-19 Patients

**DOI:** 10.3390/ijerph191912688

**Published:** 2022-10-04

**Authors:** Katarzyna Tomaszewska, Bożena Majchrowicz, Katarzyna Snarska, Donata Telega

**Affiliations:** 1 Department of Nursing, Institute of Health Protection, The Bronisław Markiewicz State Higher School of Technology and Economics, 37-500 Jarosław, Poland; 2 Department of Nursing, Institute of Social and Health Sciences, East European State Higher School in Przemyśl, 37-700 Przemyśl, Poland; 3 Department of Clinical Medicine, Medical University of Bialystok, 15-089 Białystok, Poland; 4 Institute of Health Protection, The Bronisław Markiewicz State Higher School of Technology and Economics, 37-500 Jarosław, Poland

**Keywords:** stress, work environment, SARS-CoV-2 pandemic, nurse, occupational burnout

## Abstract

COVID-19 pandemic brings many challenges to the daily work of nurses. While carrying out professional tasks for patients infected with the SARS-CoV-2 virus, nurses experience tremendous psychological pressure due to their workload in a high-risk environment. This causes severe stress and leads to occupational burnout. The purpose of this study was to assess the level of stress and occupational burnout among surveyed nurses working with patients with COVID-19. A total of 118 nurses working with patients infected with SARS-CoV-2 virus participated in the study. Among the respondents, there were 94.9% women and 5.1% men. The average age of the respondents was 38.1 +/− 2.1. The survey was conducted between April and May 2022. The research tool was a survey questionnaire, consisting of three parts: sociodemographic data and self-administered survey questionnaire containing questions about the specifics of working with COVID-19 patients. The third part was a standardized tool: the MBI Burnout Questionnaire by Christina Maslach. Participation in the study was anonymous and voluntary. Statistical analysis for independence of variables used the Chi-square test. On the other hand, coefficients based on the Phi test and Kramer’s V test, as well as non-parametric Mann–Whitney U-test (for 2 samples) and Kruskal–Wallis test (for more than 2 samples) were used to determine the strength of the relationship. During these analyses, in addition to standard statistical significance, the corresponding “*p*” values were calculated using the Monte Carlo method. The results obtained allow us to conclude that surveyed nurses working with COVID-19 patients are exposed to various stressors leading to occupational burnout. The vast majority of respondents, i.e., 90.7%, believe that stress is an integral part of the nursing profession and the average of MBI burnout among respondents was 55.67 +/− 9.77 pts., emotional exhaustion 24.74 +/− 6.11, depersonalization 12.42 +/− 2.99 and a sense of personal achievement 18.52 +/− 4.50 which means that only slightly more than half of the nurses surveyed noticed symptoms of occupational burnout themselves. The research has revealed that working with a patient who is positive for COVID-19 is a cause of stress and is related to experiencing symptoms of burnout in the group of surveyed nurses.

## 1. Introduction

At the end of January 2019, the World Health Organization (WHO) reported an outbreak of a public health threat caused by the spread of a new virus called coronavirus-2019 (COVID-19) [1,2,3]. In March 2020, a global pandemic was declared, given its international transmission affecting large numbers of people, causing significant deaths and massive social and economic disruption [4,5]. At the time, health care workers, including nursing staff who had to cope with anxiety, fear, emotional exhaustion, and other feelings, worked under tremendous physical and emotional pressure and put themselves at risk of infection while fulfilling their professional roles. As a result, the pandemic has led to a number of challenges for the nursing profession, including increased patient volumes, higher workloads, and the use of new procedures related to COVID-19 [5,6,7,8,9]. One of the negative effects of the pandemic is the deterioration of medics’ mental health. Mental problems of medical personnel, especially female nurses, are not only stress or bad emotions. Depressive states, insomnia, and anxiety are very common among them. About 50.4% of medical workers admitted to depressive episodes. Feelings of anxiety, caused by an increase in stressful situations, were experienced by up to 90% of health care workers. The most common stressors include effects of the pandemic that we are often unable to cope with, such as dealing with the death of patients whose treatment has not been successful, feeling of helplessness, and the possibility of infecting family members [10,11].

Throughout the pandemic, people experience a range of negative emotions, such as feelings of danger, uncertainty, frustration, or anger. They tend to feel sad, lonely, and confused. These emotions lead to suffering and destroy well-being, satisfaction, and enjoyment of life. They not only reduce quality of life but lead to mental health problems. The most important source of anxiety in a pandemic is, of course, the disease itself and its consequences: we fear for our own health and that of our loved ones, and these fears are often accompanied by the fear of death. Anxiety can also involve isolation, distancing, prohibition of movement, obligation to wear masks, and often limits to cognitive and social functioning [11,12,13,14,15]. Working under stress and psychosocial risks is associated with increased worker absenteeism, lost productivity, and high health and social care costs [16].

Additional problems for medical staff included onerous working conditions associated with the need to wear extra protective clothing; issues related to meeting physiological needs; temporary relocation away from families (hotel accommodations specially designed for COVID-19 hospital staff); the inevitability of confrontation with patients, their caregivers and families’ reactions to the illness; hospitalization; and onerous contact with family members caused by the suspension of visits. The situation was further exacerbated by problems that existed in the Polish health care system before the pandemic, such as staff shortages, low wages, inadequacy of the system, and neglect of key problems in previous years. All the above factors negatively affected the performance of medical personnel during the pandemic [13,17].

The time of the pandemic was not easy, which is why the support of supervisors, employees, and trusted people was so important, as mental health was more important than physical health at that moment. The worst thing that happened to medical personnel was the heckling, rejection, and stigma coming from strangers and often friends and family. Those working in single-name hospitals and infectious disease wards were most vulnerable to this phenomenon. The absurdities faced by nurses include situations in which stigma affects not only nurses but also their families and children. An additional problem on duty is the reaction of patients to not being able to get out of isolation, prohibition of visits from loved ones, and lack of contact with family causing resentment towards medical personnel [11,17].

Nurses often face tremendous psychological pressure as a result of overwhelming workloads, long hours, shift duties, and work in high-risk environments [18,19]. All this leads to the emergence of the phenomenon of occupational burnout. According to the Maslach concept, burnout is a response to excessive stress at work, which is characterized by a sense of emotional exhaustion through a negative and distanced reaction to other people and a decreased sense of competence and productivity at work [20,21,22,23]. There is a decrease in physical, emotional, and mental energy due to work-related stress, which leads to cynicism toward clients and co-workers and a low sense of self-efficacy. Burnout can occur due to work overload; lack of staff, due to value conflicts and lack of a sense of community [24,25,26]. Similarly, the World Health Organization (WHO) has defined occupational burnout as a syndrome of exhaustion, feelings of negativity, and reduced personal effectiveness due to prolonged work stress that has not been effectively treated [27,28]. Burnout syndrome does not manifest itself immediately but appears as a gradual reaction of emotional breakdown due to prolonged exposure to stress factors, leading to increased levels of dehumanization and professional dissatisfaction [29]. Over the past decade, the syndrome has become more prevalent, and in May 2019, it was recognized as an occupational hazard [30]. Because of its serious consequences, whether for staff productivity, customer satisfaction, or the reputation of the institution, occupational burnout is attracting a lot of attention. Possible reasons that make nurses particularly susceptible to it may include the extra time it takes to meet the requests of patients and families; lack of respect, teamwork, and collaboration between nurses and other healthcare professionals; and nurses’ poor skills in dealing with stressors [28].

Therefore, the mental health of nurses working with COVID-19-infected patients must be monitored and maintained during the outbreak. The services provided will be of high quality only if the work environment provides nurses with the right conditions to support them [7,11,31].

This research attempts to determine whether working with patients infected with the SARS-CoV-2 virus has an impact on stress levels and job burnout among surveyed nurses working in hospital wards.

## 2. Materials and Methods

### 2.1. Research Design

In the present study, a cross-sectional survey was conducted among nurses working with patients infected with SARS-CoV-2 virus in a hospital in Podkarpackie voivodeship in Poland, who were at work during the survey. The survey was conducted between April and May 2022.

### 2.2. Research Tools

The research tool was a survey questionnaire. It consisted of sociodemographic data and a self-administered survey questionnaire containing multiple questions on the specifics of working with COVID-19 patients. The questions in this part of the survey concerned the occurrence of stressful situations, the nature of the work (single or double shift), the department’s adaptation to working with infected patients, the provision of personal protective equipment, receiving an allowance for working with positive patients, performing swabs for SARS-CoV-2, received support, and opinions on whether mass media had an impact on the experienced level of stress. In this section, respondents were also asked to subjectively rate the level of stress they experienced on a scale of 1 to 5, where 1—meant no stress, 2—light stress, 3—more stress, 4—very high stress, and 5—stress resulting in an inability to do the job. In addition, the respondents were asked to subjectively assess their feelings about the presence of 14 symptoms that are characteristic of occupational burnout syndrome, marking their frequency. The third part was a standardized tool: the Maslach Burnout Inventory (MBI) questionnaire. It was developed by Christina Maslach in 1981. The tool was designed to assess the three components of burnout syndrome: emotional exhaustion, depersonalization, and a reduced sense of personal accomplishment. The questionnaire consists of 22 questions, which are divided into three groups, and each group deals with one of the three components of burnout. Group I includes questions 1 through 9, which deal with emotional exhaustion. Group II includes questions 10 through 14, which verify symptoms related to depersonalization, and Group III includes questions 15 through 22, which deal with a reduced sense of personal achievement. Respondents’ scores are calculated separately for each subscale, according to the key. To calculate the risk of burnout, positive responses from groups I and II and negative responses from group III should be added. The results obtained in the last scale should be interpreted taking into account the dimensions signifying professional burnout: a reduced sense of personal achievement means a high level of burnout. Participation in the survey was anonymous and voluntary.

### 2.3. Participants

The study group consisted of 118 nurses employed at the hospital. A total of 160 survey questionnaires were distributed, which accounted for 50% of the number of nurses employed; 126 questionnaires were returned, and 118 were statistically analyzed after preliminary verification of the completeness of the responses. The study group included nurses who work in the departments of internal medicine, neurology, cardiology, surgery, neurological rehabilitation, anesthesiology, and intensive care. Each respondent independently and voluntarily completed the survey questionnaire and gave written consent to participate in the study, and each respondent received information about the processing of respondents’ personal data. The consents and survey questionnaires are in the possession of the author of the paper. All distributed questionnaires were accepted and completed. Inclusion criteria for the study were a minimum of two years of working with SARS-CoV-2 infected patients and possessing a nursing profession. The exclusion criterion was the lack of consent to participate in the study and the lack of work with COVID-19 patients. The study used probability sampling, also known as random sampling. It is a selection process in which each individual in the population has the same probability (greater than 0) of being selected for the sample. The questionnaires were left in the nursing annex and after completion were personally collected by the authors of the study.

### 2.4. Statistical Analysis

The primary tests used for statistical analyses were for independence of variables the Chi-square test. On the other hand, coefficients based on the Phi test and Kramer’s V were used to determine the strength of the relationship, as well as non-parametric tests for assessing differences in Kruskal-Wallis (for more than 2 samples). During these analyses, in addition to standard statistical significance, the corresponding “*p*” values were calculated using the Monte Carlo method. The analysis was performed using the IBM SPSS 26.0 package with the Exact Tests module. All relationships/correlations/differences are statistically significant when *p* ≤ 0.05.

### 2.5. Ethical Procedures

Participation of nurses in the study was voluntary and anonymous. The study was conducted in accordance with the ethical standards set forth in the Declaration of Helsinki (64th WmA General Assembly, Fortaleza, Brazil, October 2013) and in accordance with Polish legal regulations. The study was approved by the Research Ethics Team at the Institute of Health Protection of The Bronisław Markiewicz State Higher School of Technology and Economics in Jarosław (KB/04/2022).

## 3. Results

The survey was conducted among 118 selected nurses employed at a hospital in the Podkarpackie voivodeship, Poland. The characteristics of the study group are shown in Table 1.

According to 47.5% (*n* = 56) of respondents, they feel stress at work during every duty, followed by 43.2% (*n* = 51) of respondents who chose the answer sometimes. Stress at work is rarely felt by 8.5% (*n* = 10) of respondents, and not at all by only 0.8% (*n* = 1). According to respondents, the greatest stress is felt due to the high responsibility for the health and lives of patients 72.0% (*n* = 85), followed by the large number of procedures that a nurse must perform while on duty 65.3% (*n* = 77). On the other hand, 40.7% (*n* = 48) of respondents indicated that relationship with co-workers and relationship with superiors 38.1% (*n* = 45) of respondents were stressors. According to 35.6% (*n* = 42) of respondents, it is the relationship with patients, and contact with patients’ families 34.7% (*n* = 41) that causes stressful situations. Only two respondents do not feel stress when working with people infected with the SARS-CoV-2 virus. The vast majority of respondents, i.e., 90.7% (*n* = 107), believe that stress is an integral part of the nursing profession; 49.2% (*n* = 58) of respondents said that stress at work affects their family life to a medium degree, while 29.7% (*n* = 35) of respondents believe that to a significant degree.

The nurses surveyed were asked to subjectively rate the level of stress they experienced when working with SARS-CoV-2 infected patients. The results are shown in Table 2.

Respondents were also asked whether they had SARS-CoV-2 virus testing at their workplace, and 57.6% (*n* = 68) of respondents confirmed that such testing was done regularly.

The factors that have the greatest impact on changes in personal life according to respondents are working overtime 56.9% (*n* = 67), feeling fear for their and their family’s health i.e., 55.9% (*n* = 66) and lack of free time 49.2% (*n* = 58). In addition, the same group of respondents said that the work of a nurse during a pandemic is more stressful 49.2% (*n* = 58). According to 92.4% (*n* = 109) of respondents, the required personal protective equipment was provided at the workplace, while only 25.4% (*n* = 30) of respondents said they had been trained on its proper use.

A group of 83.1% (*n* = 98) of respondents did not receive any support at their workplace from a psychologist and 48.3% (*n* = 57) of respondents received support from a ward nurse. Balanced working hours on the so-called infectious side of 3 h and on the clean side of 3 h were observed according to 64.4% (*n* = 76) of respondents, while a place to rest in the clean area was provided according to 63.6% (*n* = 75) of nurses. Furthermore, 70.3% (*n* = 83) of respondents received an allowance of 100% of their salary. Mass media have a significant impact on shaping respondents’ opinions on the level of anxiety and stress before the next duty with SARS-CoC-2 patients according to 42.4% (*n* = 50) of respondents.

Respondents were asked to indicate the symptoms of occupational burnout that they observed themselves. The detailed distribution of responses is shown in Table 3.

93.2% (*n* = 110) of respondents said that staff shortages due to COVID-19 illnesses were very common at their workplace. The resulting workload had an impact on respondents’ stress levels. Respondents who believe that staff shortages occur in the department as a result of illnesses among staff are more likely than others to feel resentment and indifference before going to work and are significantly more likely to identify negative feelings in themselves. The correlation coefficient is statistically significant and has a clear strength of association (Kramer’s V = 0.399, Chi-square = 18.809, df = 6, *p* ≤ 0.004, Monte Carlo *p* = 0.005).

The presence of symptoms of burnout was indicated by 38.1% (*n* = 45) of respondents; 39.0% (*n* = 46) of respondents believe that the topic of burnout does not concern them, while 22.9% (*n* = 27) of respondents chose the answer “I don’t know” (Table 4).

Analysis with the Kruskal–Wallis test showed that the level of MBI varied statistically significantly due to the subjective evaluation of the level of occupational burnout.

The mean of MBI burnout was 55.67 pts. +/− 9.77 (the standard deviation provides information about the dispersion of the scores (Table 5). The smaller the deviation, the more the individual scores clustered around the mean). The minimum value of burnout was 30 pts., while the maximum value was 76 pts. The higher the score, the higher the professional burnout in each area studied. This also applies to a reduced sense of personal achievement—the higher the score in this area, the higher the burnout. Analysis with the Kruskal–Wallis test showed that the level of MBI score was not statistically significantly differentiated by age and gender of the respondents.

There is only one statistically significant correlation between the three domains of job burnout, and it is characterized by significant strength of association. It is observed that higher emotional exhaustion is associated with higher levels of depersonalization (correlation coefficient of 0.667).

During the analysis, there was no statistically significant correlation between age and occupational burnout in general and considering its individual domains. The results are shown in Table 6.

Only two statistically significant correlations were shown between age and symptoms of occupational burnout. Moreover, they are characterized by weak strengths of association. It can be observed that the older age of the respondents is associated with less frequent irritability and less frequent feeling of chaos in the workplace (the correlation is positive because the higher the value in the question was, the less frequently the burnout symptom was felt by the respondents). The results are shown in Table 7.

## 4. Discussion

This study attempts to determine whether working with patients infected with the SARS-CoV-2 virus had an impact on stress levels and job burnout among surveyed nurses working in hospital wards. Respondents participating in the study indicated that the most significant factors affecting stress levels were excessive workload and high responsibility for the health and lives of patients. In our study, 47.5% of the respondents answered that they felt stress at work during each duty station, and 43.2% of the respondents chose the answer often. The average MBI burnout score was 55.67 pts. +/− 9.77, emotional exhaustion 24.74 pts. +/− 6.11, depersonalization 12.42 pts. +/− 2.99 and sense of personal achievement 52 pts. +/− 4.50, which means that only slightly more than half of the nurses surveyed noticed symptoms of job burnout in themselves.

Studies by other authors show that there is a link between burnout and depression, stress and burnout, or chronic fatigue and burnout. Burnout can develop as a chronic reaction to stress. In the Piotrowska et al. study, the mean overall MBI score was 49.27 ± 19.76 (EE = 63.56 ± 25.37, DEP = 37.2 ± 24.95, and no PA = 47.05 ± 22.04), meaning that only half of the nurses surveyed noticed burnout in themselves [32]. Shah reports that 31.5% of respondents reported a desire to change careers due to burnout, and hospital conditions and working more than 20 h a week were associated with a higher likelihood of its occurrence [26]. Lavoie-Tremblay et al. showed that high levels of chronic fatigue, poor quality of care, lower job satisfaction, and a higher desire to leave the organization were found among nurses caring for patients with COVID-19. Nurses who were poorly prepared for difficult working conditions and overwhelmed showed a higher turnover intention than those who were well prepared and in control [5]. Similar findings were obtained in their study by Kędra and Nowocień, who found that the nursing profession is associated with stressors that can be divided into factors specific to the profession and factors related to working conditions [33]. The most frequently indicated stressors at work were excessive duties, responsibility for the health of another person, and dissatisfaction and resentment of patients and their families. On the other hand, an important factor influencing the onset of burnout syndrome is the overload of professional duties [32]. Moreover, a study by Grochowska et al. showed that the main stressor among nurses and paramedics is primarily a very high level of responsibility. Nurses are overburdened by excessive demands and shift work and claim that their work is often stressful, leading to physical and mental exhaustion [34]. Dall’Ora et al. identified reduced work productivity, poor quality of care, poor patient safety, adverse events, negative patient experiences, medication errors, infections, patient falls, and intent to leave the job among the effects of burnout [21].

Respondents, when asked about the occurrence of symptoms of burnout, answered that anxiety/stress was common (48.3%), apathy was sometimes manifested by 36.4%, and headache was common in 39.0% of respondents. According to Grzelak and Szwarc, the pandemic outbreak caused a change in the perception of stress in 98.5% of respondents, and 89.2% noticed increased stress symptoms in themselves. Stress was compounded primarily by changes at the level of work organization and fear of infection and transmission of the virus from work to family. In 6.2% of respondents, thoughts of resignation or changing jobs were frequent. After a year of working in pandemic, stress remained at medium to low levels [35]. On the other hand, Szwamel et al. proved that 71.43% of respondents reported low and moderate levels of job satisfaction, while 40.85% (203) showed high and moderate levels of depersonalization. A group of 62.57% showed marked or borderline anxiety disorders, while 38.83% suffered from depression or its borderline symptoms [17].

Research on professional burnout among nurses worldwide confirms the results of the present study. The incidence of occupational burnout syndrome has been steadily increasing in the vast majority of nurses. Although EE and DP scores were higher in those who had been involved in the care of patients with COVID-19, there were no statistically significant differences in these scores compared to those who had not been involved in the care of patients with COVID-19 in the past 2 weeks. PA scores tended to be higher in those who engaged in COVID-19 care than in those who did not. Among those who engaged in COVID-19 care in the past 2 weeks, 50.0% of them experienced burnout, while burnout was reported in 9.5% of those who did not. Moreover, those who engaged in COVID-19 care were significantly more likely to experience burnout than those who did not. Healthcare worker burnout is a serious problem during a pandemic that must be addressed to ensure sustainable healthcare [36]. Sillero and Zabalegui’s results showed emotional exhaustion in 43% of nurses, depersonalization in 21%, and decreased personal fulfillment in 53% of respondents. The degree of overall burnout was moderate [37]. A review of articles found that among nurses during the COVID-19 pandemic, 57.14% indicated moderate burnout and 42.86% indicated high levels of burnout [3]. In Kelly, Gee, and Butler’s study, 54.0% of respondents suffer from moderate burnout, with emotional exhaustion scores increasing by 10% and cynicism scores by 19% after one year. The effect of burnout on organizational turnover was significant [24,28]. Feleke et al. also found that burnout among nurses employed at Addis Ababa’s private hospitals was highly prevalent [29]. Borges et al. conducted a cross-sectional study among nurses in Portugal, Spain, and Brazil, which found that about 42% of nurses showed medium to high levels of burnout, with no differences between countries (Portugal and Brazil 42%, Spain 43%). A comparative analysis showed higher levels of burnout among young nurses and those working shifts [29]. This is also a phenomenon among Nigerian nurses, who experience medium to high levels of emotional exhaustion, medium levels of depersonalization, and high levels of personal achievement [25].

In our study, 39.0% of respondents believe that the topic of job burnout does not concern them, 38.1% chose “yes”, while 22.9% of respondents chose “I don’t know”. Most respondents believe that before going to work they are accompanied by indifference, i.e., 21.2%; 19.5% of people show curiosity, and reluctance is found in 16.1% of people. The study has revealed that the older age of the subjects was associated with less frequent irritability and less frequent feelings of chaos in the workplace. The level of MBI is not statistically significantly differentiated by sociodemographics, as confirmed by studies by other authors [38].

One of the most typical consequences of occupational burnout, for the advanced stage of development of this syndrome, is the thought of changing jobs or professions. This issue was addressed by the question of the survey questionnaire, aimed at obtaining information on how often such thoughts appeared in the female respondents. Only 16.0% of female respondents admitted that they had never considered this option in the context of their professional work. Of the remaining 84.0% of female respondents, as many as 9.0% constantly think about it, and another 8.0% even think about it several times a month. In contrast, a study conducted in China found that nurses there who had higher self-esteem characteristics reported less emotional exhaustion, which translated into higher professional performance. The authors concluded that improving coping strategies may be helpful in preventing burnout among nurses, thereby increasing professional effectiveness [34].

The results of our own and other authors’ studies prove that burnout syndrome among nurses is a global phenomenon. The increasing demands on the nursing profession are essential in preparing them to cope with problems at work, avoid burnout, and reduce the negative impact on health. Prevention of occupational stress and burnout, which is becoming an increasingly common health risk for those working with COVID-19 patients, is a major challenge mainly for the occupational health service. Improving psychosocial working conditions and reducing the stress experienced by employees contributes to maintaining and improving their health, as well as maintaining their ability to work. This is particularly important in a coronavirus pandemic situation. In addition, a friendly environment, as well as the fact that the employer cares about the health of employees, promotes greater commitment to work [11,39]. The above aspects aimed at preventing burnout should be implemented at the level of education of nursing students, among whom the stress of the clinical learning environment is a recurring problem [40].

### Limitations of the Study

Data collection took place in a group of nurses working in one health care facility over a certain period of time, so the results of the study and the conclusions drawn from it cannot be generalized. In addition, nurses had an opportunity to exchange their opinions during the survey. The surveyed group was very small compared to the total number of professionally active nurses in Poland (at the end of 2021, there were 299,640 professionally active nurses in the country). It is necessary to conduct further multi-center studies to generalize the results and implement recommendations for management.

## 5. Conclusions

The survey of hospital-employed nursing staff providing services in units for patients infected with the COVID-19 virus provides insight into the impact of the SARS-CoV-2 pandemic on nurses’ stress levels and occupational burnout.

Results of the study revealed that nurses working with COVID-19 patients are exposed to various stressors that may lead to professional burnout. The study showed that according to the opinions of the surveyed group of nurses, working conditions with COVID-19 positive patients are related to experiencing symptoms of professional burnout. Due to the shortage of nursing staff, which is a significant problem for many health care units, measures should be developed and implemented to reduce the incidence of burnout among this professional group.

## Figures and Tables

**Table 1:** Characteristics of the study group.

Variable		Respondents (*n* = 118)	
Gender	Female	112	94.9%
	Male	6	5.1%
Age (years)	20–30	42	35.6%
	31–40	27	22.9%
	41–50	30	25.4%
	>50	19	16.1%
Education	Medical high school	14	11.9%
	Bachelor of Science in Nursing	49	41.5%
	Master of Science in Nursing	55	46.6%
Work experience (years)	2–5	35	29.7%
	5–10	24	20.3%
	11–20	20	16.9%
	>20	39	33.1%
Shift work	No	11	9.3%
	Yes	107	90.7%

**Table 2:** How would you rate the level of stress experienced when working with SARS-CoV-2 infected persons on a scale of 1 to 5?

Level of Stress	Frequency (*n*)	Percentage (%)
1	11	9.3
2	42	35.6
3	46	39.0
4	18	15.3
5	1	0.8
Total	118	100.0

**Table 3:** How would you rate the level of stress experienced when working with SARS-CoV2 infected persons on a scale of 1 to 5?

No.	Have You Noticed Any of the Following Symptoms of Occupational Burnout Since the Beginning of the Pandemic? (*n* = 118)	Always		Often		Sometimes		Rarely		Never	
		*n*	%	*n*	%	*n*	%	*n*	%	*n*	%
1.	Anxiety	8	6.8	57	48.3	39	33.1	9	7.6	5	4.2
2.	Apathy	3	2.5	31	26.3	43	36.4	25	21.2	16	13.6
3.	Boredom	3	2.5	28	23.7	44	37.3	27	22.9	16	13.6
4.	Anger	9	7.6	41	34.7	44	37.3	22	18.6	2	1.7
5.	Impulsiveness	7	5.9	36	30.5	38	32.2	33	28.0	4	3.4
6.	Headache	7	5.9	46	39.0	42	35.6	17	14.4	6	5.1
7.	Dizziness	5	4.2	15	12.7	47	39.8	23	19.5	28	23.7
8.	Vomiting/nausea	0	0	8	6.8	29	24.6	24	20.3	57	48.3
9.	Irritability	10	8.5	43	36.4	39	33.1	20	16.9	6	5.1
10.	Workaholism	9	7.6	29	24.6	36	30.5	21	17.8	23	19.5
11.	Heart problems	4	3.4	19	16.1	29	24.6	26	22.0	40	33.9
12.	Insomnia	9	7.6	34	28.8	36	30.5	20	16.9	19	16.1
13.	Chaos at the workplace	23	19.5	41	34.7	25	21.2	20	16.9	9	7.6
14.	Bad atmosphere in the team	11	9.3	41	34.7	31	26.3	23	19.5	12	10.2

**Table 4:** Respondents’ subjective opinion on the occurrence of occupational burnout.

Have You Observed the Occurrence of Symptoms of Occupational Burnout?	Average	Median	Average Rank	*n*	Standard Deviation	Minimum	Maximum
Yes	62.18	64.00	83.03	45	7.13	48	76
I don’t know	56.22	57.00	61.57	27	8.82	36	72
No	48.98	50.00	35.26	46	8.06	30	65
**Total**	55.67	56.00		118	9.77	30	76
**Kruskal–Wallis H**	44.582						
***p***	<0.001						
***p* (Monte Carlo)**	<0.001						

**Table 5:** MBI score—threat of occupational burnout.

	MBI Score—Threat of Occupational Burnout (22–88 pts.)	Emotional Exhaustion (9–36 pts.)	Depersonalization (5–20 pts.)	Reduced Sense of Personal Achievement (8–32 pts.)
*n*	118	118	118	118
Average	55.67	24.74	12.42	18.52
Median	56.00	26.00	12.00	19.00
Standard deviation	9.77	6.11	2.99	4.50
Minimum	30	9.00	5.00	8.00
Maximum	76	36.00	20.00	32.00

**Table 6:** Occupational burnout vs. age of respondents.

			MBI Total—Threat of Occupational Burnout (22–88 pts).	Emotional Exhaustion 1–9 (9 to 36 pts).	Depersonalization 10–14 (5 to 20 pts).	Sense of Personal Achievement 15 to 22 (8 to 32 pts.)
Spearman’s rho	Age	Correlation coefficient	−0.010	0.134	0.022	−0.171
		Significance (two-tailed)	0.913	0.149	0.811	0.063
		*n*	118	118	118	118

**Table 7:** Prevalence of burnout symptoms vs. age of respondents.

Have You Noticed Any of the Following Symptoms of Job Burnout Since the Beginning of the Pandemic?			Age
Spearman’s rho	Anxiety and stress	Correlation coefficient	0.138
		Significance (two-tailed)	0.135
		*n*	118
	Apathy	Correlation coefficient	0.001
		Significance (two-tailed)	0.992
		*n*	118
	Boredom	Correlation coefficient	0.134
		Significance (two-tailed)	0.148
		*n*	118
	Anger	Correlation coefficient	0.107
		Significance (two-tailed)	0.248
		*n*	118
	Exuberance	Correlation coefficient	0.162
		Significance (two-tailed)	0.080
		*n*	118
	Headache	Correlation coefficient	0.101
		Significance (two-tailed)	0.275
		*n*	118
	Dizziness	Correlation coefficient	0.026
		Significance (two-tailed)	0.781
		*n*	118
	Vomiting or nausea	Correlation coefficient	0.076
		Significance (two-tailed)	0.412
		*n*	118
	Irritability	Correlation coefficient	0.182
		Significance (two-tailed)	0.049
		*n*	118
	Workaholism	Correlation coefficient	−0.007
		Significance (two-tailed)	0.943
		*n*	118
	Heart problems	Correlation coefficient	−0.096
		Significance (two-tailed)	0.302
		*n*	118
	Insomnia	Correlation coefficient	−0.059
		Significance (two-tailed)	0.523
		*n*	118
	Chaos in the workplace	Correlation coefficient	0.218
		Significance (two-tailed)	0.018
		*n*	118
	Bad atmosphere in the team	Correlation coefficient	0.175
		Significance (two-tailed)	0.058
		*n*	118
